# Supplementary material for: Are carbon emissions trading and green financial instruments synergistic? -Comprehensive quantitative research based on content analysis
Source: PLoS One. 2024 Mar 7;19(3):e0298601. doi: 10.1371/journal.pone.0298601 (PMC10919601; doi:10.1371/journal.pone.0298601)
Supplement: S1 Data — (ZIP) [file pone.0298601.s001.zip › Data/地级市Z_F工作报告.zip!/╝¬┴╓/░╫╔╜╩╨/└┤╫╘╣1⁄2╓┌║┼í╛┬φ┐╦╩2╛▌═°í┐_2011.docx]

一、回顾“十一五”：团结奋进，攻坚克难，白山实现历史新跨越

　　——经济总量快速扩充。预计五年完成地区生产总值1540亿元、全口径财政收入145.6亿元、地方级财政收入78.5亿元、全社会固定资产投资1580亿元、规模以上工业增加值664.9亿元，年均分别增长19.8%、27.6%、36.2%、47.8%和24.8%。三次产业结构由2005年的16.2:51.4:32.4调整到10:58:32。2010年单位GDP综合能耗比2005年降低22%，二氧化硫提前一年完成减排任务，化学需氧量排放总量减少10%，市中心区空气质量良好天数达到318天。

　　——改革开放成效显著。支付改制成本18.1亿元，完成108户国有企业和111户厂办大集体企业改革，非公有制经济占GDP比重由53%提高到70%。政府机构改革、国库集中收付改革和集体林权制度改革主体任务按期完成，农村综合改革扎实推进。累计引进域外资金741.3亿元，年均增长61.8%。实现外贸进出口总额12.7 亿美元，年均增长20%。

　　——城乡面貌焕然一新。交通建设累计投资91亿元，新建公路1026公里，总里程达到6375公里。长白山机场建成通航，环长白山旅游公路等相继建成，营城子至松江河高速公路、靖宇至松江河铁路等开工建设。市区投资72亿元，完成了道路、桥梁、供水、供热、供气管网改造等一批市政工程。“六路安居工程”完成954万平方米，安置居民13.8万户。改造农村泥草房2.95万户。市民、农民人均居住面积分别达到26平方米、22.6平方米。

　　——人民生活明显改善。累计新增城镇就业15.6万人、转移农村剩余劳动力13.3万人。发放社会保障、社会救助资金43.8亿元。城镇参加养老保险、失业保险和基本医疗保险覆盖率分别达到95%、93%和91%。实现社会消费品零售总额498.8亿元，年均增长19.7%。城镇居民人均可支配收入、农民人均纯收入分别增加8168元和2577元。成功迈进中国十大生态旅游城、全国双拥模范城、省级卫生城和园林城行列，社会事业全面进步。对口援建四川黑水县洛多乡灾后恢复重建任务提前一年完成。

　　——民主法制不断加强。 “五五”普法圆满结束，全社会的法律意识进一步增强。认真贯彻民族区域自治法，促进了民族地区经济社会发展和各民族团结进步。

　　2010年，是全面完成“十一五”计划的收官之年，经济社会继续保持良好的发展态势。

　　——坚持大发展、快发展，综合实力明显增强。预计全年实现地区生产总值440亿元，增长18%。全口径财政收入43.2亿元、地方级财政收入25.3亿元，分别增长18.9%和27.8%。财政支出突破百亿元大关，增长31%，人均支出全省第一，创历史最好水平。固定资产投资530亿元，增长30%。规模以上工业增加值210亿元，增长25%。农业总产值71亿元，增长12.6%。第三产业增加值141亿元，增长12%。社会消费品零售总额140亿元，增长18%。引进域外资金251亿元，增长30%。实际利用外资1.3亿美元，增长20%。实现外贸进出口总额2.2亿美元，增长31%，其中出口1.8亿美元，增长28.6%。

　　——坚持高投入、快建设，发展环境明显优化。投资20.5亿元，全面实施畅通、绿美、亮化、净化、安居和暖房子工程，向江大桥、浑江大桥主体完工，新建17条街路。长白山广场、翠柏广场建设进展顺利，南岭广场基本建成，市区绿化覆盖率达到27%。日处理5万吨的污水处理厂一期投入使用。改造供热主干网和支线管网33.3公里。启动了碱厂沟河、月牙河区域综合治理工程。城市棚户区、煤矿棚户区改造完成68.1万平方米，安置居民10089户。5万平方米城镇廉租房主体完工。新农村建设投资6.5亿元，实施项目1248个，新建18个高标准示范村和20个整体推进村。解决了3.5万农村人口安全饮水问题，完成了7675户农村泥草房和危旧房改造。交通建设投资36.2亿元，营城子至松江河高速公路、朝长线东岗至长白段公路、三道沟至集安界边防公路进展顺利。新修农村公路230公里。鹤大高速公路白山段、白山至长春、白山至临江、松江河至长白高速公路列入省“十二五”建设规划，松江河至长白铁路正式立项。

　　——坚持惠民生、促和谐，人民生活水平明显提高。科技成果转化取得重大突破，施慧达药业获中国专利金奖提名和吉林省科技进步一等奖。白山市被命名为 “中国松花砚文化产业基地”，浑江区被命名为“中国金属镁产业示范基地”。“白山方大” 、“爽风” 百叶窗、“露水河”木屑板被评为中国驰名商标，“抚松人参”被认定为中国地理标志商标，靖宇“西洋参”获国家农业部地理标志保护产品。全部免除10万名城市和农村义务教育阶段学生学杂费。新型农村合作医疗制度不断完善，城市社区卫生服务覆盖率达100%。146个自然村完成广播电视“村村通”工程。在第十六届省运会上分别获金牌总数和团体总分第五名。人口自然增长率控制在0.12‰。全年新增就业3.9万人、下岗失业人员再就业2.3万人。启动了“五七家属工”养老保险参保续保试点。全市基本养老、失业、医疗、工伤、生育保险参保人数分别达到20.3万人、11.5万人、90.9万人、19.1万人和19.3万人。筹资2000万元设立“陈云教育基金”，扶持救济贫困学生完成学业。城乡低保、助残、助医、助孤等工作得到全面加强。城镇居民人均可支配收入和农民人均纯收入达到16840元、5924元，分别增长12%和11.5%。实现非正常访进京“零登记”，保持了信访局势稳定。全力推进警务信息化和平安白山建设，综合破案率居全省前列。加强煤矿、非煤矿山、危险化学品、道路交通、消防、食品药品等专项安全整治，事故发生率整体下降34.7%，连续31年实现无重大森林火灾。

　　——坚持抗大洪、救大灾，重建家园取得重大胜利。全市共争取救灾资金和物资1.02亿元，接受社会各界捐款4847万元。按照“六个确保”的要求，全市投入恢复重建资金23.4亿元，完成了29817户水毁房屋、71所学校、44个卫生院（所）、21个社会福利机构维修重建任务，修复水毁公路79条663公里、城市供水管道72公里、农村饮用水设施124处，重建堤防7公里，195户企业、4131个大棚恢复生产，复垦农田6954公顷，全部完成年内灾后重建任务。

　　——坚持重规范、高效率，政府自身建设明显加强。自觉接受人大的法律监督和政协的民主监督，共办理人大代表议案3件、建议43件和政协委员提案79件，办结率均达到100%。清理政府规范性文件63件，行政审批项目全省最少。村民自治和社区居民自治得到巩固和提高。快速反应和应急处置能力不断提高。

　　二、展望“十二五”：凝心聚力，拼搏进取，全力建设富民强市新白山

　　根据市委五届十三次全会部署，今后五年政府工作的总体要求是：坚持以邓小平理论和“三个代表”重要思想为指导，深入贯彻落实科学发展观，以实现经济大提速为主题，以加快转变经济发展方式为主线，以富民强市为目标，坚持生态立市、产业强市，实施投资拉动、项目带动、创新驱动战略，统筹推进新型工业化、生态城镇化和农业现代化建设，培育壮大“三五二”产业，以增量带动结构优化，以创新促进转型升级，以发展保障民生改善，加强资源和环境保护，繁荣发展社会各项事业，打造吉林东部新的经济增长极，努力建设富裕开放、文明和谐的新白山。

　　经济发展的主要目标是：努力实现“五个翻番、五个领先、五个突破”。到2015年，地区生产总值、财政收入、规模以上工业增加值、全社会固定资产投资、进出口总额五项指标翻一番半左右；人均地区生产总值、财政收入、城镇居民可支配收入、农民纯收入、社会保障覆盖面五项指标全省领先；力争全市GDP突破1200亿元，人均GDP突破9万元，全社会固定资产投资突破1000亿元，全口径财政收入突破120亿元，地方级财政收入突破80亿元，发展速度高于全省平均水平，迈进全省较发达城市行列。实现上述目标，我们必须全力建设富民强市的新白山。

　　——致力于产业升级，全力打造实力白山。坚持产业强市，区域经济一体化发展，推动“352”产业优化升级，提升经济核心竞争力。到2015年，全市工业总产值突破1800亿元，年均增长22%，三次产业比重调整到7：53：40。

　　第一，努力建设“三大基地”。一是建设东北重要能源基地。到2015年，煤炭产量达到1500万吨以上、煤化工产品50万吨以上，电力装机容量366万千瓦以上，能源产业实现增加值占全市GDP比重达到18.5%。二是建设吉林矿产冶金生产基地。到2015年，矿产冶金产业实现增加值占全市GDP比重达到10%。三是建设吉林林木加工基地。到2015年，实木复合地板产能达到2700万平方米、人造板产能100万立方米、家具50万件（套），林产工业增加值占全市GDP比重7%以上。

　　第二，努力打造“三大名城”。一是打造国际矿泉城。到2015年，矿泉饮品产能达到1000万吨，中高档矿泉饮品产值占50%以上，矿泉水产业增加值占GDP比重达到6.5%。二是打造国际生态旅游城。到2015年，接待游客1000万人次，旅游业总收入占GDP比重10%以上。三是打造中国特产名城。到2015年，全市人参精深加工达到50%以上，人参产业增加值占GDP比重达到5%。做大医药产业，到2015年，医药产业增加值占GDP比重达到6.5%。做大特色农产品产业，到2015年，特色农产品产业增加值占GDP比重达到6.5%。

　　第三，努力构建“两大中心”。一是建设吉林东部现代物流中心。到2015年，物流业增加值占GDP比重6%以上。二是建设长白山文化创意产业中心。到2015年，建设3-5个文化创意产业集聚区、1处奇石文化城，打造吉林省东部区域性文化中心。

　　——致力于品位提升，全力打造魅力白山。坚持提升品位与管理并重，优化空间布局，彰显城市特色，建成宜居宜业的山水园林城。

　　第一，高起点规划城市。着力构建“三核一带”的城乡空间架构。打造60万人口的长白山特色山水园林城；建成30万人口的抚松旅游新城；建成20万人口的靖宇矿泉城。到2015年，全市城镇化率由68%提高到77%以上。

　　第二，高标准建设城市。加大城市建设投入，在市中心区建成6个居住区、3个工业区、2个仓储区、6处公共停车场。加快采煤沉陷区治理改造。建设道路立体交叉口4处、城区主干道79条。人均拥有公共绿地面积10平方米，绿化覆盖率达到33%。

　　第三，高投入发展交通。到2015年，公路总里程达到7500公里，新建高速公路362公里、铁路303公里，实现市通高铁、县县通高速公路和铁路，成为吉林东部重要交通枢纽。

　　——致力于改革开放，全力打造活力白山。进一步深化改革，全方位扩大开放，不断增强经济发展的动力与活力。

　　第一，深化改革激发活力。健全现代市场体系，加快发展要素市场、资本市场及中介组织。创新公共财政体系，健全公共财政体制。深化行政管理改革，打造全省行政审批事项最少、收费标准最低、办事效率最高的地区。

　　第二，扩大开放创造活力。加强与通化、丹东共赢合作，建设白（山）-通（化）-丹（东）经济带。五年引进域外资金2475亿元，实际利用外资10亿美元，完成外贸进出口总额22.6亿美元，年均分别增长25%、20%和25%。

　　第三，自主创新增添活力。发明专利年度授权量进入全省前三名，科技成果转化率达到30%以上。争创中国驰名商标3-5个，吉林省著名商标8-10个。

　　——致力于绿色发展，全力打造生态白山。努力建设资源节约型和环境友好型社会，争创国家级低碳经济示范区。

　　第一，着力优化生态环境。加强森林资源保护培育，封山育林71.2万亩、人工冠下补植造林24.7万亩、中幼林抚育99万亩。到2015年，森林面积稳定在162万公顷以上，林木蓄积量增加200万立方米，森林覆盖率保持在83.2%以上。

　　第二，着力发展循环经济。到2015年，工业固体废弃物综合利用率达到65%，粉煤灰综合利用率达到85%，水资源重复利用率高于80%，单位GDP综合能耗降低16%。

　　第三，着力提升环境质量。综合开发利用餐厨废弃物，主要城镇污水处理率70%以上，生活垃圾无害化处理率60%以上，松花江流域出境水质断面达到地表水二类水质标准，鸭绿江浑江流域出境水质断面达到地表水三类水质标准，市中心区空气质量良好天数达到330天。

　　——致力于民生民利，全力打造和谐白山。完善公共保障体系，促进城乡协调发展，社会事业更加繁荣，人民生活更加殷实。

　　第一，农村环境明显改善。新建农村公路230公里，农村广播电视覆盖率达到95%以上，农村安全饮水达到100%，彻底消灭泥草房。实现农民住房砖瓦化、农村公路硬质化、通讯便捷化、饮水安全化、庭院整洁化、村庄秀美化、管理民主化。

　　第二，百姓幸福指数大幅提升。城镇新增就业17.5万人，下岗失业人员再就业10万人，农村劳动力转移14万人，基本实现充分就业。社会保障体系更加完善，实现城镇居民养老保险、失业保险、医疗保险、工伤保险、生育保险和新型农村养老保险全覆盖。改善城乡居民居住条件，让全市人民住有所居。

　　第三，社会事业长足发展。促进城乡教育均衡发展，大力发展职业教育，全面提高基础教育水平。不断完善公共卫生和医疗卫生服务体系，提高群众健康水平。着眼于打造文化名城，大力发展文化体育事业，加强城乡公共文化服务体系建设，继续保持群众体育和竞技体育在全省的优势地位。充分发挥广播电视宣传作用，营造良好的舆论氛围。稳定低生育水平，提高出生人口素质。全面贯彻党的民族宗教政策，促进少数民族地区经济社会发展。加强精神文明建设，进一步提高城乡文明水平。

　　第四，平安白山建设切实加强。深化“六五”普法和依法治理，切实维护公平正义，创建全省最平安城市，让人民群众生活得更加和谐安全。深入开展双拥共建活动，完善党政军警民合力治边机制，创建全国最稳定边境城市。全力抓好安全生产，确保人民生命财产安全，让人民群众安居乐业。

　　三、2011年工作安排：重点突破，全面提速，再创白山发展新篇章

　　2011年是“十二五”的开局之年，做好今年工作事关未来五年发展。主要预期目标是：全市地区生产总值增长23%，全社会固定资产投资增长25%，全口径财政收入、地方级财政收入均增长18%，城镇居民人均可支配收入、农民人均纯收入分别增长15%和13%，城镇登记失业率控制在4.6%以内，单位GDP综合能耗降低3.5%。

　　实现上述目标，今年政府要突出抓好八个方面工作、着力实施七大民生工程。

　　——突出投资拉动，在扩充经济总量上谋求新突破。全年计划安排3000万元以上重点项目495项，工业投资额占60%以上。加快推进152个续建项目，开工建设257个新项目，积极抓好重大项目前期准备。

　　——突出提速增效，在工业经济运行质量上实现新提升。完成规模以上工业总产值850亿元、工业增加值256亿元，均增长22%。实现利润20亿元，增长25%。一是扶优促强扩总量。力争1户企业产值突破30亿元、9户企业突破10亿元、24户企业突破5亿元。市直工业总产值力争突破100亿元，增长25%。二是立足特色建园区。投资8.6亿元，完善园区基础设施。加快推进八宝循环经济工业园百万吨焦化、镁工业园天安镁业二期等项目建设。开工建设包装工业园、“双丰”工业园。三是狠抓要素促保障。实施工业技改项目347项，完成技改投资200亿元，增长25 %。实现新产品产值65亿元，增长30%。四是节能减排提质量。全力抓好70户重点耗能企业和30项重点节能技改项目的跟踪调度，大力发展循环经济和节约型项目。

　　——突出增收致富，在新农村建设上再上新台阶。农业总产值实现82亿元，增长15%。重点实施四项工程：一是农业产业化工程。全市专业乡镇达到15个、专业村70个、特色产业示范大户500个，省级、市级龙头企业分别发展到38户和100户。调整种植结构，大力发展特色农业，新增经济作物10万亩。二是人参产业振兴工程。重点抓好40个人参生产小区建设，人参GAP种植面积达到2000万平方米。人参产业实现产值60亿元，增长50%。三是特色牧业工程。建设特种野猪产业化加工园区和20个生产基地，完成特种野猪肉绿色食品认证，全市特种野猪饲养量达到15万头。四是新农村建设“221”工程。重点抓好20个示范村、20个整体推进村建设，完成100个村“四清四改”任务。

　　——突出规模效益，在加快旅游三产发展上迈出新步伐。全年实现第三产业增加值180亿元，增长28%。一是加快推进旅游业发展。完成鸭绿江沿线旅游综合服务区规划，加快抚松仙人桥温泉城、白山湖度假区和抚松旅游新城建设，启动龙山湖度假区、江源温泉休闲会馆、十六道沟风景区建设。长白山国际旅游度假区行政及会议中心、长白山商业中心、文化中心及医院等配套设施按期竣工。规范定点一批旅游宾馆酒店，提高旅游从业人员服务水平。积极开发松花石、长白玉、黑陶等具有长白山特色的旅游纪念品。探索旅游线路经营拍卖，新增旅行社5家以上。全年接待游客350万人次，实现旅游业总收入37亿元，分别增长15%和25%。二是大力发展现代服务业。重点抓好长白山山货市场改造、长白山家具建材城二期工程建设。完善农村商品流通网络和社区服务功能，开通100户农家连锁店。培育金融保险、商务中介、娱乐会展、文化创意等现代服务业，推进“三网融合”，加快“数字白山”建设。三是努力扩大消费。重点提高城乡中低收入群体收入水平，扩大中等收入群体规模，提高消费能力。着力培育农村、社区、住房、休闲旅游、文化娱乐、宾馆餐饮、通讯和商业等八大重点消费领域，社会消费品零售总额突破168亿元，增长20%。

　　——突出功能品位，在建设山水园林城市上塑造新形象。一是坚持规划先行。将江源区纳入市中心城区规划管理，完成城区控制性规划编制。二是加快城市建设。向江大桥、浑江大桥年内竣工通车，新建、续建城市道路25条。加快长白山广场、翠柏广场建设，新增绿化面积6万平方米。新建污水主次管网24公里，新建、改建旱厕46座、压缩式垃圾中转站14座，重建趟子沟垃圾场。实施市区出口广场、堤滨园和9栋高层建筑楼体亮化。完成浑江干流南岸堤防改造、曲家营水库隧洞维修工程。加快月牙河改线治理、碱厂沟河综合治理。三是加强城市经营管理。盘活城市资产，实现城市资源配置容量效益最大化。继续开展城市管理年活动，加强对露天市场、城市巷道和居民小区环境专项整治，强化城市园林绿化、市政道路维护。进一步加强物业管理，提升小区物业管理水平。四是加快交通建设。加快营松高速公路、朝长线东岗至长白公路改造、三道沟至集安界边防公路及靖宇至松江河、泉阳至白山镇、松江河至长白铁路建设，推进鹤大高速公路白山段和松江河至长白、白山至长春、白山至临江高速公路建设，启动鹤大高速公路连接线及互通立交桥项目。公路、铁路、水路均恢复到灾前水平。五是推进生态城镇化建设。加快建设抚松新城，全面启动一期9.6平方公里土地开发，推进“七通一平”等基础建设。打造靖宇矿泉城融资平台，启动配套服务园区建设。

　　——突出开放招商，在增强发展后劲上再添新活力。务求招商引资实效。围绕“资源变资产、资产变资本、资本变资金”，推动特色资源与国内行业龙头和世界500强企业主动对接，开展多层次、专业化招商，实现资源效益最大化。抓好节庆活动，搭建招商引资平台。深化与中铝、北京控股集团、北京中矿、台湾大润发、新加坡博然、河北满城纸业等战略合作。突出项目包装谋划，抓好招商引资项目的跟踪落实，切实提高项目履约率和资金到位率。全年引进域外资金325亿元，增长30%。加快对外开放步伐。不断培育外贸新的增长点，力争新增外贸进出口经营权企业30户。加快推进对朝跨境贸易区建设。外贸进出口总额完成2.6亿美元，增长20%。

　　　　——突出增收融资，在壮大地方实力上取得新成果。开源节流促增收。充分发挥财税杠杆作用，推动项目建设，培植新增税源，增强财税后劲。强化税收征管措施，加强重点行业、重点企业、重点区域税源管理，保证主体税源持续增长。严厉打击各种偷逃骗漏税行为，堵塞税收流失漏洞，整顿和规范各种行政事业性收费、政府性基金和罚没收入，做到应收尽收。进一步推进国有资产经营，完善国有资产监管体制，建立国有资产营运平台。用足用好国家财政扶持和债务减免政策，在向上争取政策和资金方面有更大突破。硬化预算约束，大力压缩行政事业经费和一般性支出，提高资金使用效益。强化金融促发展。拓宽融资渠道，推进与省担保和东三省再担保公司的联保和再担保合作，争取再组建2家担保公司和3家以上小额贷款公司，引进域外股份制银行在我市设立分支机构，确保全市信贷增长不低于全省平均水平。加快推动东锋有色金属公司上市申报工作。

　　——突出民生保障，在和谐社会建设上再创新业绩。着力实施七大民生工程、办好27件民生实事。就业再就业工程2项：落实税收优惠政策，对符合条件的下岗再就业纳税人减免3年税收，对月营业额不足5000元的纳税人全额减免营业税、城建税、教育费附加和个人所得税；免费培训下岗失业人员1万人，新增发放小额担保贷款4000万元，城镇新增就业3.5万人，农村劳动力转移就业2000人，新增残疾人就业400人。社会保障工程5项：城市低保保障标准提高到月人均280元、农村提高到年人均1560元；“五七家属工”参加养老保险在全市铺开；城镇基本医疗保险、工伤保险实行市级统筹；市区城镇职工基本医疗报销限额由3.5万元提高到4.5万元；完善新型农村合作医疗制度，新农合参合率达到96%以上。扶弱助困工程3项：为1万名残疾人提供各类康复服务，免费为80名贫困白内障患者实施复明手术、为30名贫困肢体残疾人配装假肢；对城镇残疾人个体户参加养老保险给予50%的补贴；对考入高等院校的残疾学生和贫困残疾人家庭子女给予本科生每人3000元、专科生每人2000元的一次性就学资助。安居保障工程5项：改造煤矿棚户区、城市棚户区35.2 万平方米，新建廉租房5万平方米，启动通沟、道清、南岭和锦江路棚户区建设；管道燃气工程投资1200万元，新增供气户数1万户；改造供热管网24.9公里，改造住宅外网和楼内管网2.6公里，撤并小锅炉28台，完成既有建筑节能改造46万平方米；投资4000万元，改造667户农村泥草房；投资1.5亿元，完成矿泉湖二水源水库大坝截流及厂房建设。农民增收工程4项：新建日光温室1000亩、大棚1000亩，新增大地棚膜蔬菜3000亩；在全市6个县（市）区选择12个村，每村扶持100户，每户发展1万袋食用菌；全市扶持200个村、2万个农户，发放生产项目小额贷款不少于8亿元；突出解决好农民工工资问题，农民工投诉举报结案率、工资当期支付率均达到100%。社会事业工程7项：白山职业技术学校4600平方米实训综合楼投入使用；启动殡仪馆异地新建；市中医院竣工；市儿童福利院投入使用；投资6000万元扩建星泰农产品批发市场三期工程；深入开展群众体育活动，新增5个全民健身活动站；为10万人次提供生殖健康服务，优生工程推广普及率达到100%。平安白山工程1项：加强社区和农村网络化治安巡逻防控，新建1000个110报警标识、300个监控点。

　　在做好民生实事的同时，要认真落实国家和省教育中长期发展纲要，大力发展职业教育和学前教育。加快公共文化基础设施建设，做好松花石相关产业规划和重点项目建设。争取国家和省科技发展项目20个以上。成立以白山镁产业基地为核心的吉林省镁产业技术创新战略联盟。深化医药卫生体制改革，推进公立医院改革试点，引进外资民营医疗机构，解决好群众就医看病问题。加快广播电视数字化改造和双向化建设，全力抓好 “村村通”工程。启动“六五”普法教育，抓好社区矫正和法律援助。落实领导干部接访下访和包案制度，集中处理一批群众反映强烈的信访案件。切实抓好煤矿、非煤矿山、食品药品、道路交通、消防、森林防火、校园等重点行业和领域安全隐患的排查和专项整治，坚决杜绝重特大事故发生。建立健全气象灾害及引发的次生自然灾害防灾减灾体系，增强应对公共突发事件能力。深入开展千里边防线、真情大拥军活动，完善党政军警民合力治边机制，保持边境安全稳定。

　　四、加强自身建设：履职尽责，务实高效，全面提升政府工作新水平

　　——坚持从政有为，努力建设务实创新政府。始终把求真务实作为政府工作的永恒要求。全力打造学习型机关和研究型队伍，注重学习新知识、研究新情况、积累新经验，吃透上情，熟悉下情，善于用战略的思维、开放的视野、改革的办法来解决发展中的问题，切实做到学习文件、领会政策、指导实践。强力推进工作创新，瞄准经济转型、产业升级、城市发展、生态建设等重点工作全力攻坚，政府各部门每年推出1-2项创新举措，使政府工作形成若干亮点和特色，带动全市经济社会创新发展。坚持以科学的机制推进政府工作，推行项目化管理工作法，在目标任务、工作标准、责任主体上实施项目化清单式管理，做到工作谋划重长远、工作推动重细节、工作落实重成效，确保各项工作统筹推进。

　　——坚持施政有效，努力建设服务高效政府。始终把群众满意作为政府工作的永恒追求。按照市委开展“作风建设年”要求，切实转变政府职能，强化全体部门都是服务部门、全体岗位都是服务岗位、全体公务员都是服务员意识，积极倡导“5+2”、“白+黑”精神，政府各部门都要服务发展、服务民生，争做项目局、产业局和招商局，全面推行“一线工作法”，下工地、包企业，招客商、包项目，做到贴近基层抓服务、深入一线促发展。实行公开承诺制度，每个部门要将年度重点目标任务，在媒体公开承诺，接受社会监督。加大绩效评估考核力度，落实交办、督办、催办、查办制度，推行具体工作限时制、工作失误追究制和审批超时默许制，坚决制止行政不作为、乱作为和互相推诿扯皮现象发生，努力做到人人高效率、事事快节奏、件件求实效，确保机关运转更加规范协调、服务更加快捷高效。

　　——坚持执政有章，努力建设法治诚信政府。始终把依法行政作为政府工作的永恒主题。狠抓机关制度建设，政府各部门要对各项制度进行全面清理修订和完善规范，用制度管权、管事、管人，用程序合法促进工作合规。强化监督检查和行政问责，做到履责必实、失责必究、问责必严。完善民主决策机制，对涉及发展和民生的重大事项实行法律论证、民主决策、专家咨询、公开听证制度，推进政府决策程序化、民主化、科学化。加强民主法制建设，主动接受市人大的法律监督和市政协的民主监督，自觉接受社会监督和舆论监督，切实做到协商于决策之前、监督于实施过程。完善政务公开制度，保障人民群众的知情权、参与权和监督权。进一步规范政府部门和公务人员行政行为，做到遵法规、守信用，凡承诺的事情，坚决兑现，绝不失信于社会、失信于人民。

　　——坚持廉政有力，努力建设清正廉洁政府。始终把廉洁从政作为政府工作的永恒准则。坚持标本兼治、综合治理、惩防并举、注重预防的方针，建立健全教育、制度、监督并重的惩治和预防腐败体系，切实规范行政权力运行，在财政资金使用、国有资产运营、工程建设招投标、土地使用权出让、行政审批等重点领域，建立严格的监督制度和权力制约机制，从源头上阻止“权力寻租”问题的发生。深入开展民主评议政行风和软环境“双评”活动，坚决纠正损害群众利益的不法行为。旗帜鲜明地弘扬正气、理直气壮地反对歪风、毫不手软地惩治腐败，切实做到有权必有责、用权受监督、违法要严惩，以良好的形象聚民心、促发展。
